# Analysis of a Simulation Model to Estimate Long-term Outcomes in Patients with Nonalcoholic Fatty Liver Disease

**DOI:** 10.1001/jamanetworkopen.2022.30426

**Published:** 2022-09-13

**Authors:** Jagpreet Chhatwal, Ozden O. Dalgic, Wanyi Chen, Sumeyye Samur, Emily D. Bethea, Jade Xiao, Chin Hur, Kathleen E. Corey, Rohit Loomba

**Affiliations:** 1 Institute for Technology Assessment, Massachusetts General Hospital, Boston; 2 Harvard Medical School, Boston, Massachusetts; 3 Department of Gastroenterology, Massachusetts General Hospital, Boston; 4 Georgia Institute of Technology, Atlanta; 5 Columbia University, New York, New York; 6 NAFLD Research Center, University of California, San Diego, La Jolla

## Abstract

**Importance:**

Quantitative assessment of disease progression in patients with nonalcoholic fatty liver disease (NAFLD) has not been systematically examined using competing liver-related and non–liver-related mortality.

**Objective:**

To estimate long-term outcomes in NAFLD, accounting for competing liver-related and non–liver-related mortality associated with the different fibrosis stages of NAFLD using a simulated patient population.

**Design, Setting, and Participants:**

This decision analytical modeling study used individual-level state-transition simulation analysis and was conducted from September 1, 2017, to September 1, 2021. A publicly available interactive tool, dubbed *NAFLD Simulator*, was developed that simulates the natural history of NAFLD by age and fibrosis stage at the time of (hypothetical) diagnosis defined by liver biopsy. Model health states were defined by fibrosis states F0 to F4, decompensated cirrhosis, hepatocellular carcinoma (HCC), and liver transplant. Simulated patients could experience nonalcoholic steatohepatitis resolution, and their fibrosis stage could progress or regress. Transition probabilities between states were estimated from the literature as well as calibration, and the model reproduced the outcomes of a large observational study.

**Exposure:**

Simulated natural history of NAFLD.

**Main Outcomes and Measures:**

Main outcomes were life expectancy; all cause, liver-related, and non–liver-related mortality; and cumulative incidence of decompensated cirrhosis and/or HCC.

**Results:**

The model included 1 000 000 simulated patients with a mean (range) age of 49 (18-75) years at baseline, including 66% women. The life expectancy of patients aged 49 years was 25.3 (95% CI, 20.1-29.8) years for those with F0, 25.1 (95% CI, 20.1-29.4) years for those with F1, 23.6 (95% CI, 18.3-28.2) years for those with F2, 21.1 (95% CI, 15.6-26.3) years for those with F3, and 13.8 (95% CI, 10.3-17.6) years for those with F4 at the time of diagnosis. The estimated 10-year liver-related mortality was 0.1% (95% uncertainty interval [UI], <0.1%-0.2%) in F0, 0.2% (95% UI, 0.1%-0.4%) in F1, 1.0% (95% UI, 0.6%-1.7%) in F2, 4.0% (95% UI, 2.5%-5.9%) in F3, and 29.3% (95% UI, 21.8%-35.9%) in F4. The corresponding 10-year non–liver-related mortality was 1.8% (95% UI, 0.6%-5.0%) in F0, 2.4% (95% UI, 0.8%-6.3%) in F1, 5.2% (95% UI, 2.0%-11.9%) in F2, 9.7% (95% UI, 4.3%-18.1%) in F3, and 15.6% (95% UI, 10.1%-21.7%) in F4. Among patients aged 65 years, estimated 10-year non–liver-related mortality was higher than liver-related mortality in all fibrosis stages (eg, F2: 16.7% vs 0.8%; F3: 28.8% vs 3.0%; F4: 40.8% vs 21.9%).

**Conclusions and Relevance:**

This decision analytic model study simulated stage-specific long-term outcomes, including liver- and non–liver-related mortality in patients with NAFLD. Depending on age and fibrosis stage, non–liver-related mortality was higher than liver-related mortality in patients with NAFLD. By translating surrogate markers into clinical outcomes, the NAFLD Simulator could be used as an educational tool among patients and clinicians to increase awareness of the health consequences of NAFLD.

## Key Points

**Question:**

What are the long-term outcomes associated with nonalcoholic fatty liver disease (NAFLD) by age and stage of fibrosis?

**Findings:**

This decision-analytic modeling study that simulated the life course of 1 000 000 patients with NAFLD estimated 10- and 20-year competing liver- and non–liver-related mortality and cumulative incidence of decompensated cirrhosis and hepatocellular carcinoma by patient age and fibrosis stage at diagnosis. The simulation tool, dubbed the *NAFLD Simulator,* is publicly available and could serve as an educational tool for health care practitioners and their patients and increase public awareness of NAFLD.

**Meaning:**

The findings of this study provide important data to patients and clinicians for understanding the associations between surrogate end points and long-term adverse outcomes of NAFLD.

## Teaser

This decision analytical modeling study estimates long-term liver-related, non–liver-related, and overall mortality outcomes in a simulated patient population with nonalcoholic fatty liver disease by fibrosis stage.

## Introduction

Nonalcoholic fatty liver disease (NAFLD) is the most common cause of chronic liver disease worldwide, with an estimated prevalence ranging from 25% to 40%.^1,2,3^ In parallel with the burgeoning prevalence of obesity, the prevalence of NAFLD has continued to increase in the US. Nonalcoholic steatohepatitis (NASH), an aggressive form of NAFLD, can progress to decompensated cirrhosis and hepatocellular carcinoma (HCC). NAFLD is currently the second most common indication for liver transplantation in the US. Among candidates for liver transplant, NAFLD is the fastest increasing cause of HCC.^4^ NAFLD is associated with a substantial economic burden, incurring $103 billion in direct medical costs each year in the US.^5^

Effective treatments for NAFLD and NASH are lacking. Current options include lifestyle interventions (eg, diet, exercise), control of metabolic syndrome, and limited off-label pharmacotherapy.^6^ Weight loss can be highly effective in treating NASH,^7^ but very few patients succeed in maintaining a healthy weight in the long term.^8^ Bariatric surgical treatment has been associated with improved or completely resolved steatohepatitis and fibrosis,^1^ and improved clinical outcomes^9^; however, it is not widely used as an intervention for NASH. Several pharmacological treatments for NASH are under development, but none has yet been approved for use.^10^

Because NAFLD is a slowly progressive disease, ongoing clinical trials use surrogate markers as primary end points. Therefore, it is important that patients and clinicians understand the associations between surrogate end points and long-term adverse outcomes. For instance, what is the risk of HCC or mortality from competing liver- and non–liver-related causes associated with each NAFLD fibrosis stage? How would a patient’s prognosis change if their NAFLD fibrosis score was reduced by 1 stage? Answering these questions using clinical trials or observational studies could be prohibitively expensive and take decades to complete. While earlier studies provide data on all-cause and/or liver-related mortality,^11,12,13,14,15,16,17^ to our knowledge, quantitative assessment of disease progression in patients with NAFLD has not been systematically examined using competing risk models incorporating both liver-related and non–liver-related mortality.

The primary objective of this study was to develop a mathematical model of long-term outcomes in NAFLD, including competing liver-related and non–liver-related mortality, associated with the different fibrosis stages of NAFLD. We further developed an interactive online tool, the NAFLD Simulator, to make these results available for educational use.

## Methods

The study was exempt from institutional review board review because it used only publicly available data and was not human participants research. Informed consent was not necessary because simulated individuals were used. We followed the Consolidated Health Economic Evaluation Reporting Standards (CHEERS) reporting guideline.

### Model Overview

**Figure 1.**
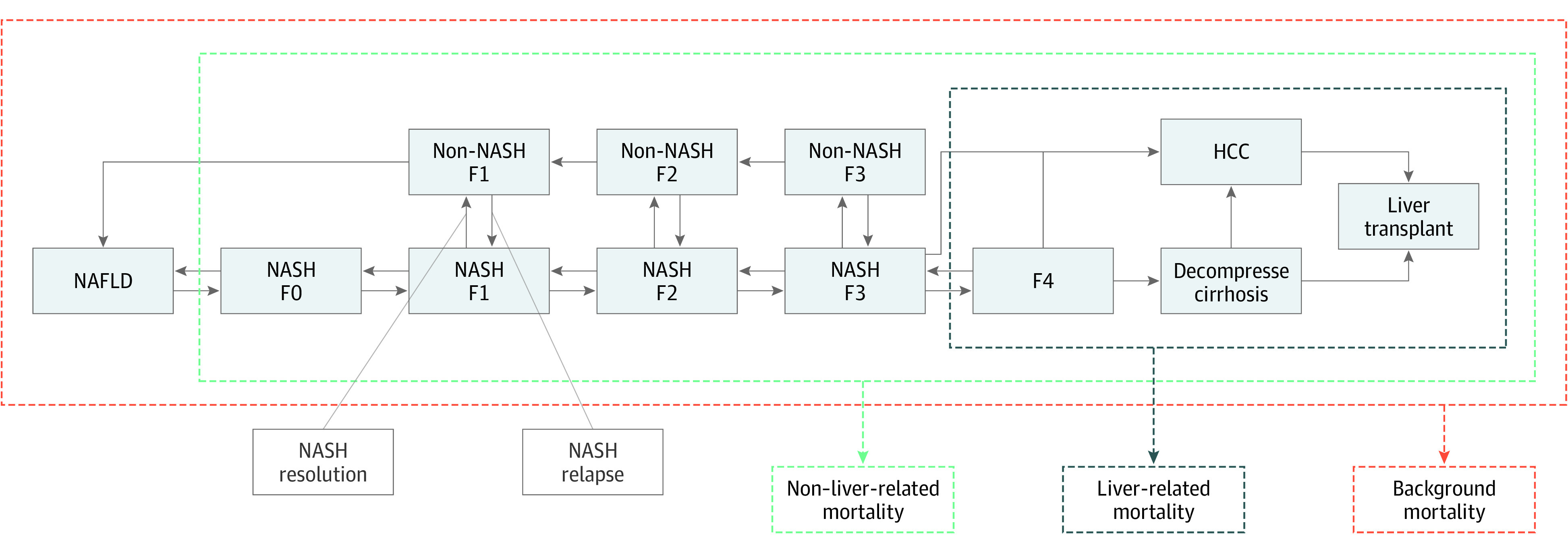
Model Schematic of the Natural History of Nonalcoholic Fatty Liver Disease (NAFLD) Each box represents a patient health state. Arrows indicate possible transitions between states. The patient can transition between different health states, including NAFL (ie, simple steatosis), NAFLD-related fibrosis states F0 to F4, decompensated cirrhosis, hepatocellular carcinoma (HCC), and liver transplant. Stages F1 to F3 are further stratified into nonalcoholic steatohepatitis (NASH) and non-NASH states. The model incorporates 3 causes of mortality: liver-related mortality, non–liver-related mortality, and background mortality. Patients’ disease in NAFLD fibrosis stages could progress or regress; these progression and regression rates were informed by either published studies or calibration.

We developed a microsimulation model that replicates the natural history of NAFLD for each patient. The patient can transition between different health states, including NAFL (ie, simple steatosis), NAFLD-related fibrosis states F0 to F4, decompensated cirrhosis, HCC, and liver transplant (Figure 1). Stages F1 to F3 are further stratified into NASH and non-NASH states. We incorporate 3 causes of mortality: liver-related mortality, non–liver-related mortality (from cardiovascular events), and background mortality. The model reproduced the outcomes of a large observational study by Hagstrom et al.^13^ The analysis was conducted from September 1, 2017, to September 1, 2021.

### Baseline Population

We simulated 1 million patients with characteristics similar to those in the NASH Clinical Research Network study.^18^ At baseline (ie, at the time of hypothetical diagnosis), patients in the F1 to F3 stages could have NASH resolution, based on published studies.^19^ The simulated patient population included 1 000 000 patients with a mean (range) age of 49 (18-75) years, including 66% women.

### NAFLD Natural History

The natural history of NAFLD was defined using the following health states: NAFL, NAFLD-related fibrosis stages F0 to F4, decompensated cirrhosis, HCC, and liver transplant (Figure 1). Owing to the slow progression of NAFLD, we chose 1 year as the cycle length. Patients’ disease in NAFLD fibrosis stages could progress or regress; these progression and regression rates were informed by either published studies or calibration.^20,21,22,23,24,25,26^ Patients could also experience NASH resolution, and those with NASH resolution could relapse; NASH resolution and relapse rates were extracted from clinical trial data.^27^ Patients with F3, F4, and decompensated cirrhosis could develop HCC. Patients with decompensated cirrhosis and HCC were classified as eligible for liver transplantation, with transplantation rates extracted from published studies using United Network for Organ Sharing data.^28,29,30,31^

We accounted for 3 types of mortalities: liver-related mortality, non–liver-related mortality (including cardiovascular events or extrahepatic malignant neoplasm), and background mortality. Patients in stages F4 and above have a higher risk of liver-related mortality in the model. Additionally, all NAFLD-associated health states (ie, excluding NAFL) were subject to non–liver-related mortality, dependent on a patient’s disease stage, sex, and age. We estimated liver- and non–liver-related mortality rates from published studies,^24,32,33,34^ and extracted background mortality from the US Life Tables.^35^

Some progression and regression rates (liver-related mortality for F4; non–liver-related mortality for F0-F4) were not directly available from published studies. These rates were estimated by calibrating our model-estimated patient survival (ie, 20-year survival by fibrosis stage at diagnosis) to reported values from a large observational study by Hagstrom et al.^13^ We used the simulated annealing algorithm to search for sets of parameter values that minimize total error between the model outcomes and reported survival.^36^ We repeated this process 1000 times, each time starting from a different initial value to generate 1000 sets of calibrated parameter values and corresponding outcomes. These sets were used to characterize the uncertainty in our estimations. Details of the calibration procedure and validation of model-estimated survival with the reported outcomes by Hagstrom et al^13^ are presented in the eAppendix in the Supplement. The full list of extracted and calibrated model parameters are presented in eTable 1 in the Supplement.

### Statistical Analysis

Our model estimated the 10- and 20-year cumulative incidence of advanced sequalae of NAFLD, as well as background, liver-related, and non–liver-related mortality by age and fibrosis stages at diagnosis using liver biopsy. For each health state, we further estimated overall survival up to 20 years, life expectancy, and survival time. We generated 95% uncertainty intervals (UIs) for the outcomes to account for uncertainty from calibration (eAppendix in the Supplement).

We developed a publicly available, interactive tool, NAFLD Simulator,^37^ using the Shiny package in R statistical software version 3.5 (R Project for Statistical Computing). The tool allows users to specify a patient profile defined by age, sex, and NAFLD fibrosis score, then provides model-estimated long-term outcomes in a graphic format. Outcomes include the cause of death (liver related, non–liver related, or background), overall survival, cumulative risks of developing decompensated cirrhosis and HCC, and probability of receiving a liver transplant.

## Results

Using the decision-analytic model, we generated outcomes for different hypothetical patients defined by their age and fibrosis stage at diagnosis. We validated the model estimated outcomes with data from an observational study.

### Model Validation

**Figure 2.**
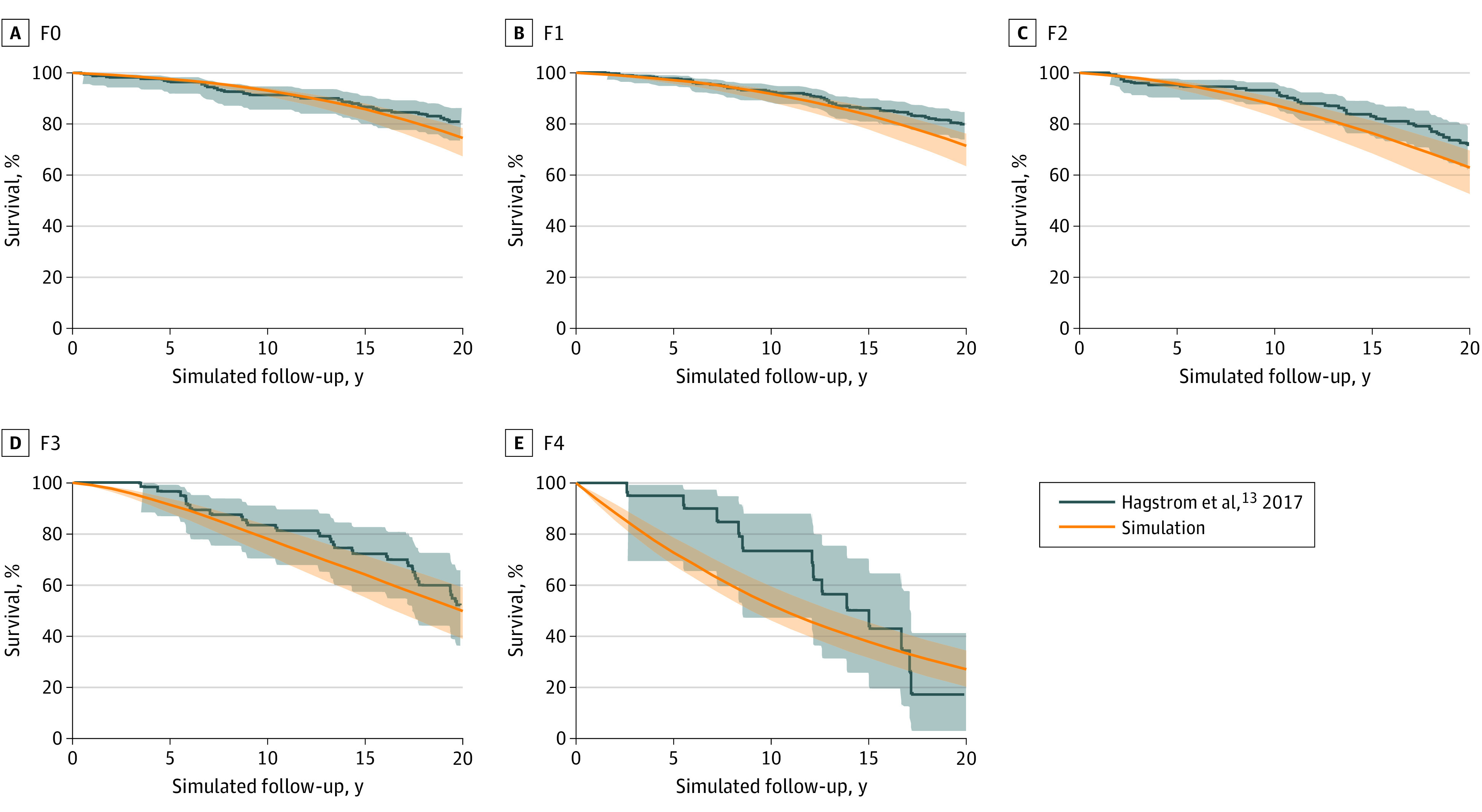
Model Validation by Comparison With Published Outcomes We compared our model-estimated long-term survival results outcomes from a simulated population cohort stratified by fibrosis states with those from Hagstrom et al.^13^

Using our model, we simulated the population reported by Hagstrom et al^13^ and generated 20-year patient survival by fibrosis stage at diagnosis. Our model-estimated overall survival closely matched the reported outcomes in the study by Hagstrom et al^13^ (Figure 2). The overlapping curves show that the model accurately estimated stage-specific Kaplan-Meier survival curves for patients with F0 through F4 for up to 20 years.^13^ We independently validated estimated all-cause mortality for patients with cirrhosis using data from a study by Simon et al.^16^

### Survival

**Figure 3.**
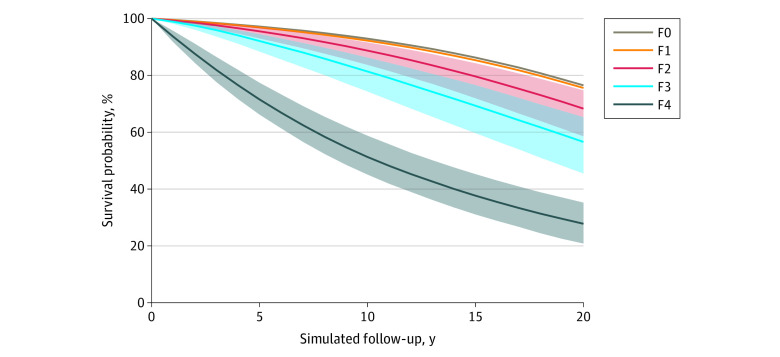
Survival in Patients Aged 49 Years With Nonalcoholic Fatty Liver Disease–Related Fibrosis Stage Shaded regions represent the 95% uncertainty intervals generated from probabilistic sensitivity analysis.

For a patient aged 49 years (the mean age), the model-estimated 10-year survival was 92.9% (95% UI, 89.8%-94.1%) for stage F0, 92.2% (95% UI, 88.4%-93.9%) for stage F1, 88.7% (95% UI, 82.3%-91.9%) for stage F2, 81.4% (95% UI, 72.9%-86.9%) for stage F3, and 51.3% (95% UI, 44.2%-59.4%) for stage F4 (Figure 3). Of note, the largest drop in survival (30.1 percentage points) was observed between stages F3 and F4. The life expectancy of patients aged 49 years was 25.3 (95% CI, 20.1-29.8) years for those with F0, 25.1 (95% CI, 20.1-29.4) years for those with F1, 23.6 (95% CI, 18.3-28.2) years for those with F2, 21.1 (95% CI, 15.6-26.3) years for those with F3, and 13.8 (95% CI, 10.3-17.6) years for those with stage F4 at baseline (ie, at the time of diagnosis). The decrease in life expectancy was 7.3 years between stages F3 and F4.

### Liver and Non–liver-related Mortality

**Figure 4.**
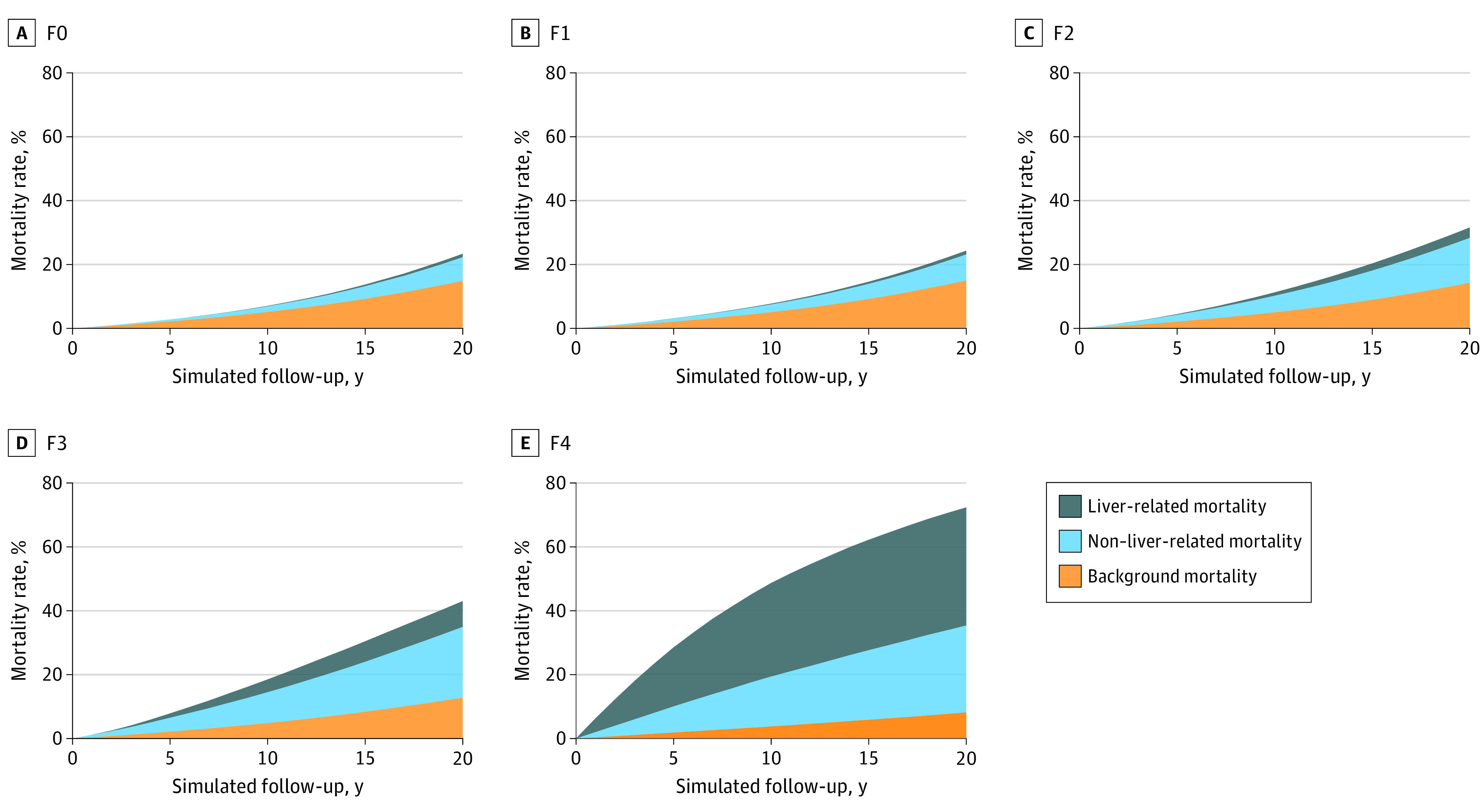
Mortality in Patients Aged 49 Years With Nonalcoholic Fatty Liver Disease–Related Fibrosis Stages F1 to F4 As fibrosis stage increased, the odds of dying from liver disease increased.

Figure 4 shows liver-related, non–liver-related, and background mortality by fibrosis stage in patients aged 49 years. The estimated 10-year liver-related mortality was 0.1% (95% UI, <0.1%-0.2%) in stage F0, 0.2% (95% UI, 0.1%-0.4%) in F1, 1.0% (95% UI, 0.6%-1.7%) in stage F2, 4.0% (95% UI, 2.5%-5.9%) in stage F3, and 29.3% (95% UI, 21.8%-35.9%) in stage F4 patients (Figure 4; eTable 2 in the Supplement). The higher the fibrosis stage, the higher the odds of death from liver disease. Compared with stage F0, 10-year liver-related mortality was larger by a factor of 2 in stage F1, by a factor of 10 in stage F2, by a factor of 40 in stage F3, and by a factor of 293 in stage F4. Of note, 10-year liver-related mortality in stage F4 was 7.3-fold higher than in stage F3. eTable 2 in the Supplement presents 20-year outcomes for NAFLD patients by disease stage.

The estimated 10-year non–liver-related mortality was 1.8% (95% UI, 0.6%-5.0%) in stage F0, 2.4% (95% UI, 0.8%-6.3%) in stage F1, 5.2% (95% UI, 2.0%-11.9%) in stage F2, 9.7% (95% UI, 4.3%-18.1%) in stage F3, and 15.6% (95% UI, 10.1%-21.7%) in stage F4 patients (eTable 2 in the Supplement). The higher the fibrosis stage, the higher the odds of non–liver-related mortality. Compared with stage F0, 10-year non–liver-related mortality was greater by a factor of 1.3 in stage F1, by a factor of 2.9 in stage F2, by a factor of 5.4 in stage F3, and by a factor of 8.7 in stage F4. Additionally, 10-year non–liver-related mortality in stage F4 was 1.6-fold higher than in stage F3.

In stages F0 to F3, 10-year non–liver-related mortality was higher than liver-related mortality; whereas in stage F4, 10-year liver-related mortality was substantially higher than non–liver-related mortality. The largest increase in mortality (both liver and non–liver) was observed when patients progressed from stage F3 to stage F4, indicating an imperative need for effective treatments and interventions to halt progression at stage F3 and aid regression from stage F4.

### Advanced Sequelae

The cumulative incidences of decompensated cirrhosis and HCC by baseline fibrosis stage in patients aged 49 years are presented in the eFigure in the Supplement. The estimated 10-year cumulative incidence of decompensated cirrhosis was 0.08% (95% UI, 0.05%-0.1%) in stage F0, 0.15% (95% UI, 0.1%-0.25%) in stage F1, 0.7% (95% UI, 0.5%-1.0%) in stage F2, 2.7% (95% UI, 2.1%-3.4%) in stage F3, and 17.2% (95% UI, 15.9%-18.6%) in stage F4 (eFigure and eTable 2 in the Supplement). Compared with stage F3, the cumulative incidence of decompensated cirrhosis in the F4 stage was 6.4 times larger. The 10-year cumulative incidence of HCC was 0.03% (95% UI, 0.02%-0.05%) in stage F0, 0.06% (95% UI, 0.03%-0.09%) in stage F1, 0.29% (95% UI, 0.19%-0.40%) in stage F2, 1.12% (95% UI, 0.88%-1.37%) in stage F3, and 7.88% (95% UI, 7.41%-8.67%) in stage F4. Compared with stage F3, the cumulative incidence of HCC in the F4 stage was 7.2-fold larger.

### Competing Liver-Related and Non–liver-related Mortality

**Figure 5.**
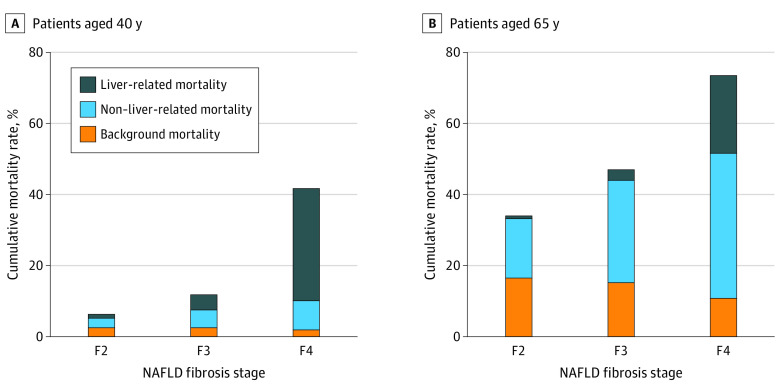
Competing Liver-Related and Non–liver-related Mortality in Patients Aged 40 Years or 65 Years With Nonalcoholic Fatty Liver Disease (NAFLD) by Fibrosis Stage

The estimated 10-year mortality (liver, non–liver, and background) in patients aged 40 years or 65 years are presented in Figure 5 and eTable 3 in the Supplement. Among patients aged 40 years, non–liver-related mortality was higher than liver-related mortality in those with stage F2 (2.7% vs 1.1%) or F3 (5.0% vs 4.3%); however, this trend was reversed in those with stage F4 (8.2% vs 31.6%) because of a higher competing liver-related mortality associated with stage F4. Among patients aged 65 years, non–liver-related mortality was higher than liver-related mortality in all stages because of higher competing cardiovascular risk in older age (F0: 6.0% vs 0.08%; F1: 8.1% vs 0.2%, F2: 16.7% vs 0.8%; F3: 28.8% vs 3.0%; F4: 40.8% vs 21.9%). Compared with patients aged 40 years, 10-year liver-related mortality was lower in those aged 65 years across all fibrosis stages; however, 10-year non–liver-related mortality was higher in patients aged 65 years, primarily because of the aforementioned higher cardiovascular risk.

## Discussion

In this decision analytic modeling study that simulated the life course of NAFLD natural history, we found a nonlinear a nonlinear increase in adverse clinical outcomes with increasing fibrosis stage. Specifically, in patients without cirrhosis, 10-year non–liver-related mortality was higher than liver-related mortality; in contrast, in patients with cirrhosis, 10-year liver-related mortality was substantially higher than non–liver-related mortality. The largest increase in mortality—both liver- and non–liver-related—was observed when patients who progressed from stage F3 to stage F4, indicating an imperative need for early recognition, effective treatments, and interventions to halt progression at the precirrhosis stage.

NAFLD represents a substantial disease burden, and our study provides several new insights into the natural history of NAFLD. While earlier studies estimated overall mortality and liver-related mortality,^12,13,14,15,16,17^ none have reported non–liver-related mortality associated with the different NAFLD stages, to our knowledge. We provide stage-specific non–liver-related mortality estimates while accounting for competing risk between liver and non–liver events. As noted in some of the earlier studies, we also found a nonlinear increase in adverse clinical outcomes with increasing fibrosis stage.^16^ In particular, when a patient progressed from stage F3 to stage F4, there was a 25% decrease in 10-year survival. Cumulative incidence of decompensated cirrhosis and HCC from the F4 stage was 7-fold higher than from the F3 stage. Therefore, preventing the progression from stage F3 to stage F4 may substantially reduce adverse outcomes, prevent the need for liver transplant in patients with NAFLD, and save health care resources.

We have also developed an interactive online tool, NAFLD Simulator, that displays our model-estimated outcomes in a graphic format. By translating surrogate marker outcomes into clinical outcome estimates in a form that can be easily understood by patients and clinicians, the NAFLD Simulator could be used as an educational tool to increase awareness of the health consequences of NAFLD among patients and health care practitioners. Users can also download simulated patient data, plots, and an executive report for a given patient profile.

The NAFLD Simulator is an accessible, easy-to-use simulation tool that could serve as an educational tool for health care practitioners and their patients and simultaneously increase public awareness of NAFLD. In future, the NAFLD Simulator could incorporate the long-term outcomes associated with new therapies. Further extensions could evaluate long-term outcomes associated with noninvasive testing methods, such as imaging and serum-based biomarkers. Our framework will be constantly updated to incorporate new evidence as it arises, thereby maintaining its relevance to the NAFLD community.

### Limitations

The findings of our study should be understood in the context of its limitations. First, our study does not account for differences in outcomes when the patient has comorbidities, such as diabetes and obesity, while it is known that long-term outcomes in different comorbidity subgroups can vary substantially. Second, our model may be limited in its applicability to the wider population because it requires patients to know their biopsy-confirmed fibrosis stage. As more data become available, our model could be extended to evaluate long-term outcomes associated with noninvasive tests, including emerging imaging and serum-based biomarkers.^38^ Third, we assumed that patients’ long-term outcomes were dependent on their current NAFLD fibrosis stage only and not on any prior change in the fibrosis score. For instance, it is possible that patients who regressed from stage F4 to stage F3 may have different outcomes than patients with stage F3 who never progressed to stage F4.

## Conclusions

In this decision analytical model study, we developed an online, interactive tool, the NAFLD Simulator, that simulates long-term outcomes in patients with NAFLD. Depending on the age and fibrosis stage at diagnosis, non–liver-related mortality can be higher than liver-related mortality in NAFLD patients. By translating surrogate marker outcomes into clinical outcomes easily understood by patients and clinicians, the NAFLD Simulator could be used as an educational tool to increase awareness of the health consequences of NAFLD among patients and health care practitioners.

## Notes

Supplement. **eAppendix.** Model Parameters, Assumptions, and Calibration **eTable 1.** Input Parameters Used to Simulate the Natural History of NAFLD in the NAFLD Simulator **eFigure.** Cumulative Incidence of NAFLD-Associated Advanced Sequelae in Patients Aged 49 Years With NAFLD-Related Fibrosis Stages F0 to F4 **eTable 2.** Mortality and Clinical Outcomes in Patients with NAFLD by NAFLD Fibrosis Stage **eTable 3.** 10-Year Mortality and Clinical Outcomes in Patients With NAFLD Aged 40 Years or 65 Years by NAFLD Fibrosis Stage
eReferences.
