# Supplementary material for: Analysis of a Simulation Model to Estimate Long-term Outcomes in Patients with Nonalcoholic Fatty Liver Disease
Source: JAMA Netw Open. 2022 Sep 13;5(9):e2230426. doi: 10.1001/jamanetworkopen.2022.30426 (PMC9471976; doi:10.1001/jamanetworkopen.2022.30426)
Supplement: Supplement. — eAppendix. Model Parameters, Assumptions, and Calibration eTable 1. Input Parameters Used to Simulate the Natural History of NAFLD in the NAFLD Simulator eFigure. Cumulative Incidence of NAFLD-Associated Advanced Sequelae in Patients Aged 49 Years With NAFLD-Related Fibrosis Stages F0 to F4 eTable 2. Mortality and Clinical Outcomes in Patients with NAFLD by NAFLD Fibrosis Stage eTable 3. 10-Year Mortality and Clinical Outcomes in Patients With NAFLD Aged 40 Years or 65 Years by NAFLD Fibrosis Stage eReferences. [file jamanetwopen-e2230426-s001.pdf]

Supplemental Online Content

Chhatwal J, Dalgic OO, Chen W, et al. Analysis of a simulation model to estimate long-term outcomes in patients with nonalcoholic fatty liver disease. *JAMA Netw Open*. 2022;5(9):e2230426. doi:10.1001/jamanetworkopen.2022.30426

eAppendix. Model Parameters, Assumptions, and Calibration

eTable 1. Input Parameters Used to Simulate the Natural History of NAFLD in the NAFLD Simulator

eFigure. Cumulative Incidence of NAFLD-Associated Advanced Sequelae in Patients Aged 49 Years With NAFLD-Related Fibrosis Stages F0 to F4

eTable 2. Mortality and Clinical Outcomes in Patients with NAFLD by NAFLD Fibrosis Stage

eTable 3. 10-Year Mortality and Clinical Outcomes in Patients With NAFLD Aged 40 Years or 65 Years by NAFLD Fibrosis Stage

eReferences.

This supplemental material has been provided by the authors to give readers additional information about their work.

© 2022 Chhatwal J et al. *JAMA Network Open.*

eAppendix. Model Parameters, Assumptions, and Calibration

Model Parameters and Assumptions

Progression and Regression Among Fibrosis Stages F0–F4

Most of the fibrosis progression and regression rates were extracted from published studies and clinical trial reports. The parameters that were not available were estimated through calibration. e**Table 1** presents values and the ranges of the annual fibrosis progression rates. We assumed that patients may progress/regress at most one stage in a single year. In the following, we use F4 and compensated cirrhosis (CC) interchangeably. Once patients progress from F4 to a higher disease stage, they cannot regress.

NASH Resolution and Relapse

We assume that patients could experience NASH resolution in fibrosis stages F0–F3 but not F4. We assumed that NASH in stages F1–F3 resolves with an annual probability of 8% (1). When NASH is resolved, fibrosis will not progress but may regress to a lower stage. For patients in the F0 stage, NASH resolution is modeled as regression to the NAFL stage. Patients in a non-NASH state can undergo NASH relapse with an annual probability of 5% (1). Relapsed patients may progress to a higher fibrosis stage. NASH status can change at most once in a year alongside fibrosis progression and regression.

Liver Transplantation

Patients with decompensated cirrhosis (DCC) and hepatocellular carcinoma (HCC) are eligible for liver transplantation. Liver transplantation probabilities are presented in **eTable 1**.

Mortality

We considered three different causes of mortality: 1) liver-related mortality (LRM), i.e., death from liver failure; 2) non-liver-related mortality (NLRM), i.e., deaths resulting from comorbidities such as cardiovascular disease; 3) background mortality (BM), i.e., deaths from any other causes.

We assumed that in a single year, patients can die from liver-related causes only if they have cirrhosis (compensated or decompensated) or had HCC. The annual risk of LRM in F4 was not available from published studies and was therefore calibrated, assuming that the annual risk of LRM in F4 was no greater than that in DCC, as described below.
Let $\mathrm{MRR}_{\mathrm{CC/DCC}}^{\mathrm{LRM}}$ denote the liver-related mortality risk ratio (MRR) of CC to DCC, i.e., the relative risk of LRM in F4 to the same in DCC. Then LRM in F4 is calculated as follows:

$$\mathrm{LRM}_{\mathrm{CC}} = 1 - (1 - \mathrm{LRM}_{\mathrm{DCC}})^{\mathrm{MRR}_{\mathrm{CC/DCC}}^{\mathrm{LRM}}}.$$

Non-liver-related mortality was assumed to be a risk for patients at any fibrosis stage (F0–F4) as well as DCC and HCC. Moreover, non-NASH F1–F3 patients were assumed to have the same annual NLRM risk as their NASH counterparts (2). We further assumed that patients' NLRM risk after transplantation is the same as their annual NLRM risk prior to transplantation.

© 2022 Chhatwal J et al. *JAMA Network Open.*

The NAFL state was not associated with additional NLRM (3, 4). NLRM for F0–F4 were not available from published studies. These were calibrated to be increasing with worsening liver fibrosis. To do this, we calibrated MRR parameters for all unknown NLRM rates for F0–F4. For example, let $\mathrm{MRR}_{\mathrm{CC/DCC}}^{\mathrm{NLRM}}$ denote the risk of NLRM in F4 relative to the same in DCC, and $\mathrm{MRR}_{\mathrm{F3/CC}}^{\mathrm{NLRM}}$ denote the risk of NLRM in F3 relative the same in F4, and so on. Using MRRs, we calculated NLRM for F0–F4 at age 49 as follows:

$$\mathrm{NLRM}_{\mathrm{CC}}^{49} = 1 - \left(1 - \mathrm{NRLM}_{\mathrm{DC}}^{49}\right)^{\mathrm{MRR}_{\mathrm{CC/DCC}}^{\mathrm{NLRM}}},$$
$$\mathrm{NLRM}_{\mathrm{F3}}^{49} = 1 - \left(1 - \mathrm{NLRM}_{\mathrm{CC}}^{49}\right)^{\mathrm{MRR}_{\mathrm{F3/CC}}^{\mathrm{NLRM}}},$$
$$\mathrm{NLRM}_{\mathrm{F2}}^{49} = 1 - \left(1 - \mathrm{NLRM}_{\mathrm{F3}}^{49}\right)^{\mathrm{MRR}_{\mathrm{F2/F3}}^{\mathrm{NLRM}}},$$
$$\mathrm{NLRM}_{\mathrm{F1}}^{49} = 1 - \left(1 - \mathrm{NLRM}_{\mathrm{F2}}^{49}\right)^{\mathrm{MRR}_{\mathrm{F1/F2}}^{\mathrm{NLRM}}},$$
$$\mathrm{NLRM}_{\mathrm{F0}}^{49} = 1 - \left(1 - \mathrm{NLRM}_{\mathrm{F1}}^{49}\right)^{\mathrm{MRR}_{\mathrm{F0/F1}}^{\mathrm{NLRM}}}.$$

The calibrated values of the MRRs are presented in e**Table 1***.* We assumed that the risk of NLRM changes with age and is proportional to background mortality at that age. Then, using the the NLRM rate at age 49 as baseline, we calculated LRM at age a as follows:

$$\mathrm{NLRM}^{a} = 1 - (1 - \mathrm{NRLM}^{49})^{\frac{\mathrm{BM}^{a}}{\mathrm{BM}^{49}}}.$$

Background mortality data were taken from the US life tables, which include mortality rates for yearly ages up to 100 (5). However, data from the Swedish life tables were used instead during calibration to replicate conditions in Sweden where the observational study by Hagstrom et al. was conducted (2). In addition, we used the Swedish life tables for the year 1990 as both Swedish calibration cohort observed and the median year of the observations was 1990 (2, 6).

© 2022 Chhatwal J et al. *JAMA Network Open.*

Model Calibration

Calibration Targets

We aimed to match model-predicted patient survival at different fibrosis stages with the reported values in a large observational study by Hagstrom et al. (2). We used a total of five survival targets (one for each fibrosis stage at diagnosis). For each survival target, we simulated a cohort with population characteristics (i.e., median age, male/female ratio) matching that from the observational studies.

Goodness-of-Fit Measure

For each survival target, we recorded the time points (years) for which our model-predicted survival fell inside or outside of the confidence interval. For any time point where our prediction was outside of the confidence interval, we assumed an error defined as the absolute distance between our prediction and the closest endpoint of the confidence interval. For time points where our predictions were inside the confidence interval, we assumed the error is zero. Let L_i^t and U_i^t denote respectively the lower and the upper bounds of the confidence interval for survival target i in year t. Let S_i^t denotes the model-predicted survival for survival target i in year t. The error at time *t* for survival target i is:

$$\gamma_i^t = \begin{cases} 0, & \text{if } L_i^t \le S_i^t \le U_i^t \\ \min\{|L_i^t - S_i^t|, |U_i^t - S_i^t|\}, & \text{otherwise} \end{cases}$$

Then, we calculated overall goodness-of-fit as the summation of model errors over the survival targets and years.

Calibration Procedure

We used simulated annealing to search for sets of parameter values that minimize total error (7). The simulated annealing algorithm starts with an initial guess of the parameter set and updates the guess in subsequent iterations (7). Because the initial value affects the search outcome, we started with 1,000 sets of initial values, where each parameter was sampled from a clinically plausible range. Then, after obtaining 1,000 sets of calibrated parameters, we updated the range for each parameter using the lower and upper 25th quartile of the sampling distribution and rerun the simulated annealing algorithm to obtain 500 sets of calibrated parameter values.

Calibration Results

We collected the results from 500 replications of simulated annealing search with different sets of initial values. These results were, as a collection, considered the calibrated parameter values. As opposed to a point estimate, a collection describes the inherent uncertainty in the calibration process.

Validation

© 2022 Chhatwal J et al. *JAMA Network Open.*

We also visually compared model-predicted 20-year patient survival by fibrosis stage at diagnosis with the reported values by Hagstrom et al. (2). We found that the model predicted curves closely overlapped with the reported values.

© 2022 Chhatwal J et al. *JAMA Network Open.*

eTable 1. Input Parameters Used to Simulate the Natural History of NAFLD in the NAFLD Simulator

Parameter	Low	Base	High	Source
**Fibrosis progression or regression (annual)**				
NAFL to F0	0.1095	0.1438[§]	0.1848	(8)
F0 to NAFL	0.0600	0.1000*	0.1400	Calibration
F0 to F1	0.1000	0.1336*	0.1700	Calibration
F1 to F0	0.0593	0.0810[†]	0.1095	(9)
F1 to F2	0.1000	0.1352*	0.1700	Calibration
F2 to F1	0.0593	0.0810	0.1095	(9)
F2 to F3	0.1000	0.1413*	0.1800	Calibration
F3 to F2	0.0593	0.0810	0.1095	(9, 10)
F3 to F4	0.1000	0.1420*	0.1800	Calibration
F3 to HCC	0.0003	0.0004	0.0005	(11)
F4 to F3	0.0860	0.1428	0.1861	(10)
F4 to DCC	0.0410	0.0411	0.0795	(12)
F4 to HCC	0.0062	0.0141[^]	0.0378	(11, 13-15)
DCC to HCC	0.0529	0.0711	0.0864	(16, 17)
From DCC to liver transplant	0.0173	0.0230	0.0288	(18-20)
From HCC to liver transplant	0.0300	0.0400	0.0500	(20, 21)
**Mortality**				
Liver-related mortality (annual)				
DCC	0.0835	0.0858	0.2395	(12)
HCC	0.4794	0.4860	0.5532	(22)
First-year after liver transplant (previously DCC)	0.0951	0.1046	0.1141	(23)
First-year after liver transplant (previously HCC)	0.1082	0.1232	0.1401	(24)
Non-liver-related mortality (annual)				
DCC	0.0224	0.0526	0.1297	(12)
HCC	0.0760	0.0800	0.1196	(22)
Liver transplant (previously DCC)	0.0224	0.0526	0.1297	(12)
Liver transplant (previously HCC)	0.0760	0.0800	0.1196	(22)
Mortality risk ratios				
Non-liver-related mortality risk ratio: F0/F1	0.2700	0.5117*	0.7500	Calibration
Non-liver-related mortality risk ratio: F1/F2	0.2400	0.4936*	0.7600	Calibration
Non-liver-related mortality risk ratio: F2/F3	0.2400	0.4931*	0.7500	Calibration
Non-liver-related mortality risk ratio: F3/ F4	0.2400	0.4957*	0.7500	Calibration
Non-liver-related mortality risk ratio: F4/DCC	0.2400	0.4821*	0.7400	Calibration
Liver-related mortality risk ratio: F4/DCC	0.2500	0.4959*	0.7400	Calibration

[§] The study reported 44-64% of the NASH patients regress to NAFL over in 3-7 years. We assumed that 54% NASH patients regress to NAFL over 5 years to calculate the transition probability from F0 to NAFL.

[†] We used "improvement in fibrosis with no worsening of NASH" statistic reported in the study as the estimates of improvement in fibrosis by one stage. Although the study cohort included only F2 and F3 patients, we assumed that same rate applies to the F1 patients.

© 2022 Chhatwal J et al. *JAMA Network Open.*

** For calibrated parameters, the base value denotes the median of all values collected from 1,000 replications of simulated annealing search.*
^ *Weighted average of HCC incidence values reported by* (11, 13-15)

Note: Calculation of annual transition probabilities: If a transition probability is not directly reported as annual probability, we used exponential conversion to calculate the annual probability by solving the following equation: $p_{reported} = 1 - e^{\lambda t_{reported}}$ *and* $p_{annual} = 1 - e^{\lambda}$ where $p_{reported}$ is the transition probability for the given period $t_{reported}$, p_{annual} is the annual transition probability, and λ is the rate of the underlying exponential distribution.

Abbreviations: NAFLD, nonalcoholic fatty liver; F0 through F4, fibrosis stage 0 through 4 (NASH Clinical Research Network system); *DCC, decompensated cirrhosis; HCC, hepatocellular carcinoma*

© 2022 Chhatwal J et al. *JAMA Network Open.*

eFigure. Cumulative Incidence of NAFLD-Associated Advanced Sequelae in Patients Aged 49 Years With NAFLD-Related Fibrosis Stages F0 to F4

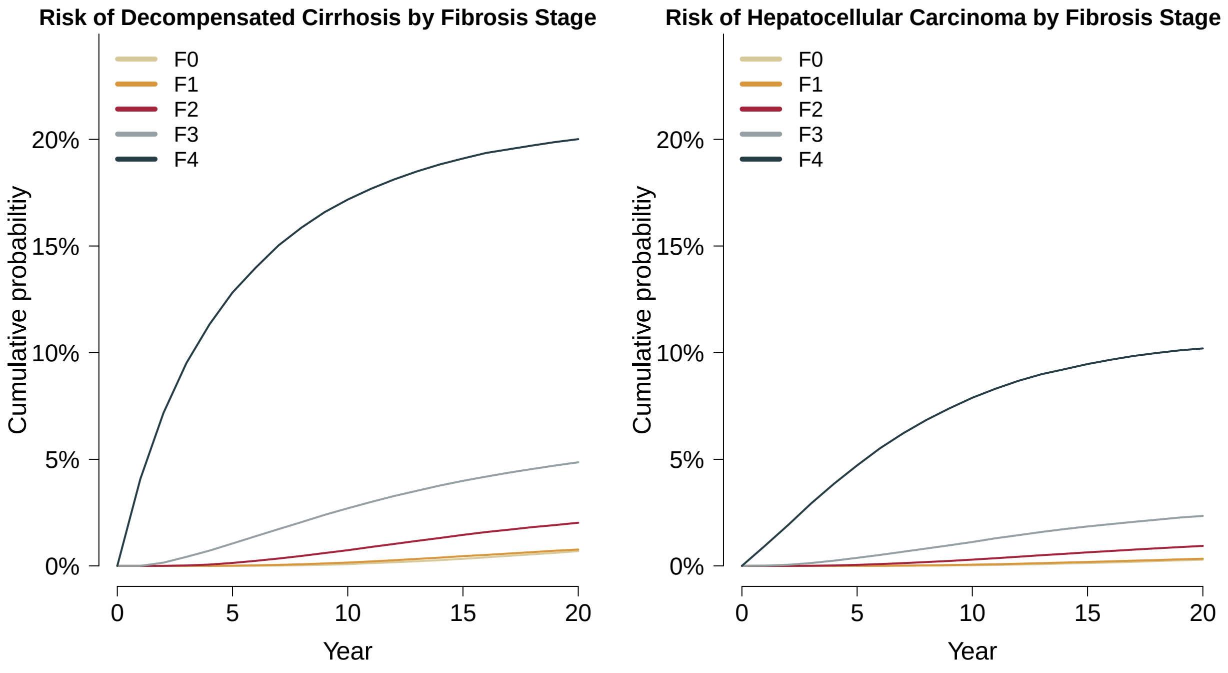

The predicted 10-year cumulative incidence of decompensated cirrhosis (DCC) was 0.08% (0.05-0.1%) in F0, 0.15% (0.1-0.25%) in F1, 0.7% (0.5-1.0%) in F2, 2.7% (2.1-3.4%) in F3, and 17.2% (15.9-18.6%) in F4 patients. Compared with F3, the cumulative incidence of DCC from the F4 stage is 6.4 times larger. The 10-year cumulative incidence of hepatocellular carcinoma (HCC) was 0.03% (0.02-0.05%) in F0, 0.06% (0.03-0.09%) in F1, 0.3% (0.2-0.4%) in F2, 1.1% (0.9-1.4%) in F3, and 7.9% (7.4-8.7%) in F4 patients. Compared with F3, the cumulative incidence of HCC from the F4 stage is 7.2 times larger.

Abbreviations: F0 through F4, fibrosis stage 0 through 4 (NASH Clinical Research Network system).

© 2022 Chhatwal J et al. *JAMA Network Open.*

eTable 2. Mortality and Clinical Outcomes in Patients with NAFLD by NAFLD Fibrosis Stage

NAFLD stage	Mortality, %			Cumulative incidence		Survival, % (UI)
	Liver-related (UI)	Non-liver-related (UI)	All-cause (UI)	DCC (per 100 person-years)	HCC (per 100 person-years)	
**10-year results**						
**NAFL**	0.009 (0.003-0.02)	0.7 (0.2-2.2)	5.9 (5.4-7.3)	0.007 (0.003-0.01)	0.002 (0.0003-0.005)	94.1 (92.7-94.6)
**F0**	0.1 (0.05-0.2)	1.8 (0.6-5.0)	7.1 (5.9-10.2)	0.08 (0.05-0.1)	0.03 (0.02-0.05)	92.9 (89.8-94.1)
**F1**	0.2 (0.1-0.4)	2.4 (0.8-6.3)	7.8 (6.1-11.6)	0.15 (0.10-0.25)	0.06 (0.03-0.09)	92.2 (88.4-93.9)
**F2**	1.0 (0.6-1.7)	5.2 (2.0-11.9)	11.3 (8.1-17.7)	0.7 (0.5-1.0)	0.3 (0.2-0.4)	88.7 (82.3-91.9)
**F3**	4.0 (2.5-5.9)	9.7 (4.3-18.1)	18.6 (13.1-27.1)	2.7 (2.1-3.4)	1.1 (0.9-1.4)	81.4 (72.9-86.9)
**F4**	29.3 (21.8-35.9)	15.6 (10.1-21.7)	48.7 (40.6-55.8)	17.2 (15.9-18.6)	7.9 (7.4-8.7)	51.3 (44.2-59.4)
**20-year results**						
**NAFL**	0.3 (0.2-0.6)	4.6 (1.7-11.6)	20.4 (17.5-26.9)	0.2 (0.1-0.4)	0.09 (0.05-0.2)	79.6 (73.1-82.5)
**F0**	1.0 (0.6-1.9)	7.4 (2.9-16.8)	23.5 (19.2-32.2)	0.7 (0.4-1.1)	0.3 (0.2-0.5)	76.5 (67.8-80.8)
**F1**	1.1 (0.7-2.0)	8.1 (3.3-17.9)	24.4 (19.8-33.3)	0.8 (0.5-1.1)	0.3 (0.2-0.5)	75.6 (66.7-80.2)
**F2**	3.2 (2.0-5.1)	14.0 (6.4-28.1)	31.7 (24.7-44.0)	2.0 (1.4-2.7)	0.9 (0.6-1.3)	68.3 (56.1-75.3)
**F3**	8.0 (5.4-11.5)	22.2 (11.0-37.7)	43.4 (33.4-56.9)	4.9 (3.7-6.1)	2.3 (1.8-2.9)	56.6 (43.1-66.6)
**F4**	37.0 (28.8-44.7)	27.2 (17.9-37.2)	72.2 (63.5-80.1)	20.0 (17.9-22.8)	10.2 (9.3-11.7)	27.8 (19.9-36.5)

Abbreviations: UI, uncertainty interval that defines the range of outcomes based on the calibration of unknown model parameters; NAFL, non-alcoholic fatty liver; F0 through F4, fibrosis stage 0 through 4 (NASH CRN system); DCC, decompensated cirrhosis; HCC, hepatocellular carcinoma.

© 2022 Chhatwal J et al. *JAMA Network Open.*

eTable 3. 10-Year Mortality and Clinical Outcomes in Patients With NAFLD Aged 40 Years or 65 Years by NAFLD Fibrosis Stage

NAFLD stage	Mortality, %			Cumulative incidence		Survival, % (UI)
	Liver-related (UI)	Non-liver-related (UI)	All-cause (UI)	DCC (per 100 person-years)	HCC (per 100 person-years)	
**Age 40**						
**NAFL**	0.006 (0.002-0.02)	0.4 (0.1-1.2)	2.9 (2.6-3.7)	0.005 (0.001-0.01)	0.002 (0.000-0.005)	97.1 (96.3-97.4)
**F0**	0.1 (0.05-0.2)	0.9 (0.3-2.5)	3.6 (3.0-5.3)	0.09 (0.05-0.1)	0.03 (0.02-0.05)	96.4 (94.7-97.0)
**F1**	0.2 (0.1-0.4)	11.2 (0.4-3.3)	4.0 (3.1-6.1)	0.16 (0.10-0.24)	0.06 (0.03-0.09)	96.0 (93.9-96.9)
**F2**	1.1 (0.6-1.8)	2.7 (1.0-6.2)	6.4 (4.6-9.6)	0.8 (0.5-1.1)	0.3 (0.2-0.4)	93.6 (90.4-95.4)
**F3**	4.3 (2.8-6.2)	5.0 (2.2-9.5)	12.0 (8.5-16.5)	2.9 (2.2-3.5)	1.2 (0.9-1.5)	88.0 (83.5-91.5)
**F4**	31.6 (23.7-38.0)	8.2 (5.3-11.5)	41.8 (33.6-47.9)	17.9 (16.8-19.4)	8.6 (8.1-9.2)	58.2 (52.1-66.4)
**Age 65**						
**NAFL**	0.006 (0.002-0.01)	2.4 (0.7-7.6)	20.2 (18.5-25.1)	0.005 (0.002-0.01)	0.002 (0.000-0.005)	79.8 (74.9-81.5)
**F0**	0.08 (0.03-0.2)	6.0 (2.0-16.5)	23.5 (19.8-33.1)	0.06 (0.03-0.1)	0.02 (0.01-0.04)	76.5 (66.9-80.2)
**F1**	0.2 (0.1-0.3)	8.1 (2.8-20.0)	25.5 (20.7-36.4)	0.1 (0.07-0.2)	0.04 (0.03-0.07)	74.5 (63.6-79.3)
**F2**	0.8 (0.4-1.4)	16.7 (6.7-35.0)	34.0 (25.0-50.4)	0.6 (0.4-0.8)	0.2 (0.1-0.3)	66.0 (49.6-75.0)
**F3**	3.0 (1.8-4.6)	28.8 (13.6-48.2)	47.0 (33.9-63.8)	2.1 (1.6-2.7)	0.8 (0.6-1.1)	53.0 (36.2-66.1)
**F4**	21.9 (15.4-28.7)	40.8 (28.0-52.6)	73.7 (63.3-82.5)	14.2 (12.6-16.1)	6.0 (5.3-6.7)	26.3 (17.5-36.7)

Abbreviations: UI, uncertainty interval that defines the range of outcomes based on the calibration of unknown model parameters; NAFL, non-alcoholic fatty liver; F0 through F4, fibrosis stage 0 through 4 (NASH CRN system); DCC, decompensated cirrhosis; HCC, hepatocellular carcinoma.

© 2022 Chhatwal J et al. *JAMA Network Open.*

eReferences

1. Neuschwander-Tetri BA, Loomba R, Sanyal AJ, Lavine JE, Van Natta ML, Abdelmalek MF, Chalasani N, et al. Farnesoid X nuclear receptor ligand obeticholic acid for non-cirrhotic, non-alcoholic steatohepatitis (FLINT): a multicentre, randomised, placebo-controlled trial. The Lancet 2015;385:956-965.
2. Hagstrom H, Nasr P, Ekstedt M, Hammar U, Stal P, Hultcrantz R, Kechagias S. Fibrosis stage but not NASH predicts mortality and time to development of severe liver disease in biopsy-proven NAFLD. J Hepatol 2017;67:1265-1273.
3. Dulai PS, Singh S, Patel J, Soni M, Prokop LJ, Younossi Z, Sebastiani G, et al. Increased risk of mortality by fibrosis stage in nonalcoholic fatty liver disease: Systematic review and meta-analysis. Hepatology 2017;65:1557-1565.
4. Singh S, Allen AM, Wang Z, Prokop LJ, Murad MH, Loomba R. Fibrosis progression in nonalcoholic fatty liver vs nonalcoholic steatohepatitis: a systematic review and meta-analysis of paired-biopsy studies. Clin Gastroenterol Hepatol 2015;13:643-654.e641-649; quiz e639-640.
5. Arias E, Heron MP, Xu J. United States life tables, 2013. 2017.
6. Life table by sex and age. Year 1960 - 2018. In.
7. Aarts E, Korst J. Simulated annealing and Boltzmann machines. 1988.
8. Perumpail BJ, Khan MA, Yoo ER, Cholankeril G, Kim D, Ahmed A. Clinical epidemiology and disease burden of nonalcoholic fatty liver disease. World J Gastroenterol 2017;23:8263-8276.
9. Younossi ZM, Ratziu V, Loomba R, Rinella M, Anstee QM, Goodman Z, Bedossa P, et al. Obeticholic acid for the treatment of non-alcoholic steatohepatitis: interim analysis from a multicentre, randomised, placebo-controlled phase 3 trial. Lancet 2019;394:2184-2196.
10. Harrison SA, Wong VW-S, Okanoue T, Bzowej N, Vuppalanchi R, Younes Z, Kohli A, et al. Selonsertib for patients with bridging fibrosis or compensated cirrhosis due to NASH: Results from randomized phase III STELLAR trials. Journal of Hepatology 2020;73:26-39.
11. Kanwal F, Kramer JR, Mapakshi S, Natarajan Y, Chayanupatkul M, Richardson PA, Li L, et al. Risk of Hepatocellular Cancer in Patients With Non-Alcoholic Fatty Liver Disease. Gastroenterology 2018;155:1828-1837.e1822.
12. Sanyal AJ, Banas C, Sargeant C, Luketic VA, Sterling RK, Stravitz RT, Shiffman ML, et al. Similarities and differences in outcomes of cirrhosis due to nonalcoholic steatohepatitis and hepatitis C. Hepatology 2006;43:682-689.
13. Orci LA, Sanduzzi-Zamparelli M, Caballol B, Sapena V, Colucci N, Torres F, Bruix J, et al. Incidence of Hepatocellular Carcinoma in Patients With Nonalcoholic Fatty Liver Disease: A Systematic Review, Meta-analysis, and Meta-regression. Clin Gastroenterol Hepatol 2021.
14. Simon TG, Roelstraete B, Sharma R, Khalili H, Hagström H, Ludvigsson JF. Cancer Risk in Patients With Biopsy-Confirmed Nonalcoholic Fatty Liver Disease: A Population-Based Cohort Study. Hepatology;n/a.
15. Vilar-Gomez E, Calzadilla-Bertot L, Wai-Sun Wong V, Castellanos M, Aller-de la Fuente R, Metwally M, Eslam M, et al. Fibrosis Severity as a Determinant of Cause-Specific Mortality in Patients With Advanced Nonalcoholic Fatty Liver Disease: A Multi-National Cohort Study. Gastroenterology 2018;155:443-457.e417.
16. Fattovich G, Giustina G, Degos F, Tremolada F, Diodati G, Almasio P, Nevens F, et al. Morbidity and mortality in compensated cirrhosis type C: a retrospective follow-up study of 384 patients. Gastroenterology 1997;112:463-472.
17. Planas R, Balleste B, Alvarez MA, Rivera M, Montoliu S, Galeras JA, Santos J, et al. Natural history of decompensated hepatitis C virus-related cirrhosis. A study of 200 patients. J Hepatol 2004;40:823-830.

© 2022 Chhatwal J et al. *JAMA Network Open.*

18. Thuluvath P, Guidinger M, Fung J, Johnson L, Rayhill S, Pelletier S. Liver transplantation in the United States, 1999–2008. Am J Transplant 2010;10:1003-1019.
19. Wong RJ, Singal AK. Trends in Liver Disease Etiology Among Adults Awaiting Liver Transplantation in the United States, 2014-2019. JAMA Network Open 2020;3:e1920294-e1920294.
20. Younossi ZM, Stepanova M, Ong J, Trimble G, AlQahtani S, Younossi I, Ahmed A, et al. Nonalcoholic Steatohepatitis Is the Most Rapidly Increasing Indication for Liver Transplantation in the United States. Clin Gastroenterol Hepatol 2021;19:580-589.e585.
21. Lang K, Danchenko N, Gondek K, Shah S, Thompson D. The burden of illness associated with hepatocellular carcinoma in the United States. J Hepatol 2009;50:89-99.
22. Younossi ZM, Otgonsuren M, Henry L, Venkatesan C, Mishra A, Erario M, Hunt S. Association of nonalcoholic fatty liver disease (NAFLD) with hepatocellular carcinoma (HCC) in the United States from 2004 to 2009. Hepatology 2015;62:1723-1730.
23. Cholankeril G, Wong RJ, Hu M, Perumpail RB, Yoo ER, Puri P, Younossi ZM, et al. Liver Transplantation for Nonalcoholic Steatohepatitis in the US: Temporal Trends and Outcomes. Dig Dis Sci 2017;62:2915-2922.
24. Younossi Z, Stepanova M, Ong JP, Jacobson IM, Bugianesi E, Duseja A, Eguchi Y, et al. Nonalcoholic Steatohepatitis Is the Fastest Growing Cause of Hepatocellular Carcinoma in Liver Transplant Candidates. Clin Gastroenterol Hepatol 2019;17:748-755.e743.

© 2022 Chhatwal J et al. *JAMA Network Open.*
